# Biological Features of Extracellular Vesicles and Challenges

**DOI:** 10.3389/fcell.2022.816698

**Published:** 2022-06-24

**Authors:** Ye Zeng, Yan Qiu, Wenli Jiang, Junyi Shen, Xinghong Yao, Xueling He, Liang Li, Bingmei Fu, Xiaoheng Liu

**Affiliations:** ^1^ Institute of Biomedical Engineering, West China School of Basic Medical Sciences and Forensic Medicine, Sichuan University, Chengdu, China; ^2^ Laboratory Animal Center of Sichuan University, Chengdu, China; ^3^ Department of Biomedical Engineering, The City College of the City University of New York, New York, NY, United States

**Keywords:** exosomes, biological feature, clinical application, drug delivery, biomarker

## Abstract

Extracellular vesicles (EVs) are vesicles with a lipid bilayer membrane on the outside, which are widely found in various body fluids and contain biological macromolecules such as DNA, RNA, lipids and proteins on the inside. EVs were once thought to be vesicles for the removal of waste materials, but are now known to be involved in a variety of pathophysiological processes in many diseases. This study examines the advantage of EVs and the challenges associated with their application. A more rational use of the advantageous properties of EVs such as composition specificity, specific targeting, circulatory stability, active penetration of biological barriers, high efficient drug delivery vehicles and anticancer vaccines, oxidative phosphorylation activity and enzymatic activity, and the resolution of shortcomings such as isolation and purification methods, storage conditions and pharmacokinetics and biodistribution patterns during drug delivery will facilitate the clinical application of EVs.

## 1 Introduction

Extracellular vesicles (EVs) are shed from most cells carrying proteins, lipids, adenosine-triphosphate (ATP), ions such as calcium, and nucleic acids. The cargos of EVs exert multiple biological functions that are critical for mother-foetus crosstalk (Cho et al., 2020), immune surveillance (Wen et al., 2017), inflammation (Sanwlani and Gangoda, 2021) and oxidative stress (Chiaradia et al., 2021), regulate a wide range of pathological processes of neurological disorders (Gratpain et al., 2021) and cardiovascular diseases (Han et al., 2021), and serve as key players in the pre-metastatic niche formation (Becker et al., 2016) and subsequent orgnotropic metastasis (Hoshino et al., 2015) of cancer. Currently, EVs can be generally classified as exosomes that are derived from multivesicular bodies (MVBs), microvesicles that are shed from the plasma membrane, or as apoptotic bodies that originate from cells undergoing apoptosis (Soekmadji et al., 2020). Specifically, exosomes are biologically active EVs with a bilayer membrane structure of approximately 30–150 nm in diameter that are secreted from living cells and carry biologically active substances contained in cells of their origin, such as nucleic acids, proteins, lipids, ATP, and metabolites, may vary largely owing to their parent cell types and the pathophysiologic status (Liang et al., 2021). Exosomes are formed from MVBs that evolved from intraluminal vesicles as inward budding of the membrane of early endosomes, and released into the extracellular space to extrude intracellular components, by fusion of the MVBs with the plasma membrane of cells (Picca et al., 2022). After release from the cell surface, exosomes can interact with the extracellular matrix or enter into recipient cells to elicit responses in the intracellular microenvironment, even travelling a long distance.

Exosomes were firstly identified in 1983 by Johnstone et al. in vesicles released from sheep reticulocytes (Johnstone et al., 1987), which were thought to be mere “waste products” of reticulocyte maturation. Studies have shown that exosomes are natural vectors present in many types of extracellular fluids that can regulate cellular stress and activate intracellular signalling pathways (Picca et al., 2022). Exosomes have been found in a variety of body fluids including plasma, serum, urine, cerebrospinal fluid, breast milk, saliva, cell culture supernatant, and others (Wang S. et al., 2021). Exosomes are capable of translocating various active biomolecules from their origin cells to the recipient cells and thus regulate the morphology and function of recipient cells, which can be beneficial or detrimental to the organisms.

**FIGURE 1:**
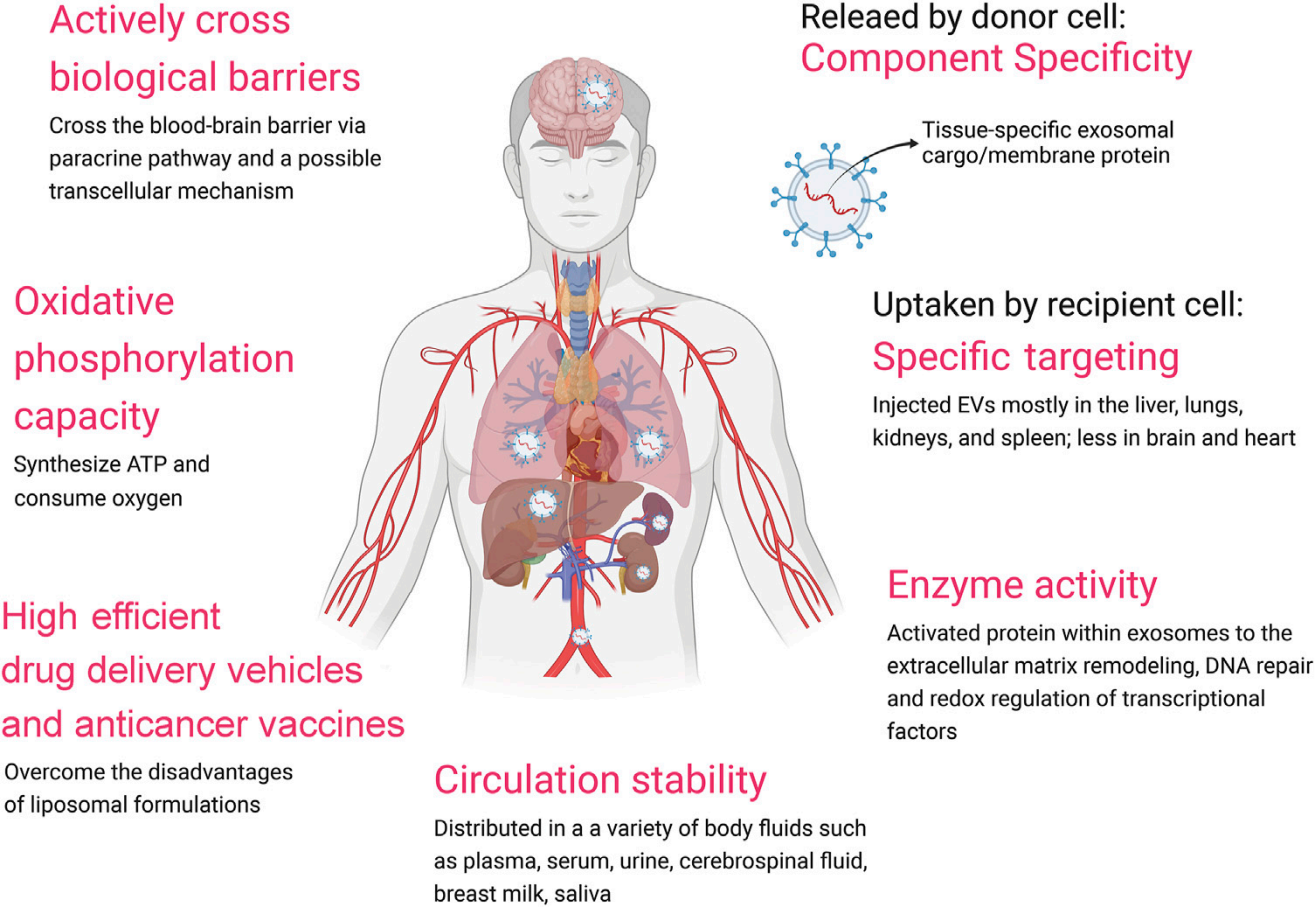
Advantages of EVs.

Recent studies have shown that EVs possess several advantages over traditional drug delivery vectors such as liposomes, including *in vivo* stability and high efficient (van der Meel et al., 2014). Furthermore, EVs are able to cross the blood-brain barrier (BBB) (Terstappen et al., 2021). EVs hold a great promise in acting as drug carriers. The heterogeneity of EVs in term of size and contents reflects the state and type of the cell of origin, and it has been suggested that EVs can be used as biomarkers for diagnosis of various diseases such as various type of cancers or even fetal sex determination (Keller et al., 2011; Kim et al., 2021). The specific EV contents including proteins, DNA, microRNAs (miRs), long non-cording RNAs (lncRNA) and circular RNAs (circRNAs) have been exploited as biomarkers (Eissa et al., 2016; Hurwitz et al., 2016; Li et al., 2021). In particular, the DNA in circulation EVs from body fluid is a promising biomaterial to replace cellular DNA for biopsy (Sharma and Johnson, 2020). EVs are high efficient drug delivery vehicles (i.e., MSC-dervied EVs) and anticancer vaccines (i.e., DC-derived EVs, and CAR-T derived EVs). However, there are currently some limitations to the clinical application of EVs, such as the lack of standards for isolation and purification, difficulty in preservation, and limited drug delivery efficiency. The International Society for EVs introduced the state of the art and current challenges in EV-based biomarker discoveries and applications and EV-based therapies (Soekmadji et al., 2020). In this mini-review, we highlight our outstanding on the biological properties of EVs (Figure 1) and the challenge of their clinical application (Supplementary Table S1), which aims to provide an important reference for the clinical application of EVs.

## 2 Biological Properties of EVs

### 2.1 Component Specificity

The content of EVs may reflect the specific characteristics and status of donor’s cells or tissues. Levels of circulating EVs have changed in the blood of patients with cancers (Peinado et al., 2012), atherosclerosis (Oggero et al., 2021), and osteoporosis (Lee et al., 2021). The protein content commonly present in EVs was revised in MISEV 2018 (Théry et al., 2018), including two categories: transmembrane or GPI-anchored proteins associated with plasma membrane and/or endosomes (such as CD63, syndecans, ADAM10, and sonic hedgehog), and cytosolic proteins recovered in EVs (such as TSG101, ALIX, flotillins-1, annexins, HSC70, HSPA8, and HSP90AB1). The proteomic profile of 426 human samples from tissue explants, plasma and other body fluids showed that the most prominent markers found in human-derived EVs were HSPA8, HSP90AB1, CD9, programmed cell death 6-interacting protein (ALIX), beta-actin (ACTB), moesin (MSN), and ras-related protein 1b (RAP1B) (Hoshino et al., 2020). EVs protein signature versican (VCAN), tenascin C (TNC) and thrombospondin 2 (THBS2) can distinguish tumors from normal tissues with 90% sensitivity/94% specificity (Hoshino et al., 2020). Proteomic characterization of EVs from a panel of 60 cancer cell lines representing nine different cancer types from the National Cancer Institute (NCI-60) showed that those EVs shared only 213 proteins, while overall, more than 6,000 proteins were unique (Hurwitz et al., 2016). A total of 1,726 proteins and 461 mRNAs were quantified in EVs derived from clear cell renal cell carcinoma, papillary renal cell carcinoma, and benign kidney cell lines. Among them, 181 proteins and 159 mRNAs were detected in all samples (Zieren et al., 2021). Shared proteins represent factors involved in biogenesis, whereas unique proteins reflected the characteristics of their cell of origin and were therefore proposed as biomarkers.

EVs have specific surface protein molecules, which are ideal non-invasive biomarkers for disease diagnosis. For example, exosomes in the urine of prostate cancer patients contain biomarkers that can be used for prostate cancer diagnosis (Nilsson et al., 2009). In tumor, exosomal VEGF is a novel biomarker for anti-angiogenesis resistance (Zeng et al., 2019). Endothelial cell (EC)-derived exosomes carry high levels of proteins that promote atherosclerosis and may be used as diagnostic biomarkers for cardiovascular disease (Goetzl et al., 2017). Exosomal mRNAs, *ALPL*, and *CXCR2* were identified as potentially useful biomarkers for acute myocardial infarction (He et al., 2021).

Exosomal miRs can be used as functional diagnostic biomarkers. Based on the syber-green PCR array, the three most significantly upregulated miRs including miR-15b, miR-636, and miR-34a were identified in urine exosomes of patients with diabetic kidney disease (Eissa et al., 2016). By using the next-generation sequencing technology combined with bioinformatics analysis, 544 upregulated and 518 downregulated exosomal miRs in acute myocardial infarction patients were identified, and miR-6718-5p and miR-4329 can be used as potential diagnostic biomarkers (Chen S. et al., 2021). Using high-throughput sequencing analysis, it was identified a total of 63 miRNAs such as miR-130a-5p were differentially expressed in exosomes from bone marrow mesenchymal stem/stromal cells (MSCs) between diabetic ulcer patients and healthy adults (Liu P. et al., 2021). Moreover, the presence of lncRNA and circRNAs in plasma exosomes might provide biomarkers for early diagnosis of various diseases, including cancers, metabolic diseases, neurodegenerative diseases, cardiovascular diseases, autoimmune diseases, and infectious diseases (Li et al., 2021).

Exosomal DNA is an excellent biomaterial to replace cellular DNA for biopsy because of its longer length and higher stability compared to circulating tumor cell DNA (Sharma and Johnson, 2020). Mutations of EV-derived DNA reliably reflect the mutational state in the tumor of origin (García-Silva et al., 2020). For example, EGFR mutation was presented in plasma exosomes from lung cancer (Krug et al., 2018; Purcell et al., 2021) and cerebrospinal fluid EVs from glioblastoma (Figueroa et al., 2017), and KRAS mutation was presented in plasma exosomes from pancreatic cancer (Allenson et al., 2017) and colorectal cancer (Lucchetti et al., 2021). Some EV-derived DNA is tissue-specific. For example, exudative seroma contained more circulating exosomes than plasma, and exosomes derived from exudative seroma and plasma displayed different proteomic profiles (García-Silva et al., 2019). Thus, plasma, urine, cerebrospinal fluid and bronchoalveolar lavage fluid have been explored for liquid biopsy tests.

Given the presence of specific cargos in exosomes reflecting their originated cells, there is a great interest in identifying exosomes contents as biomarkers and currently there were 61 clinical trials (status in completed, recruiting, and enrolling) (Supplementary Table S1). From these clinical trials thus far, one can see that the changes in characteristics of blood (plasma or serum) and urine circulating exosomes including level, size, concentration and/or subgroups are expected to be biomarkers of colorectal cancer, osteosarcoma, non-small cell lung cancer, pancreatic cancer, elite endurance athletes, intracerebral hemorrhage, type 2 diabetes, and gestational diabetes. Exosomal cargos including RNA, miRNAs, autophagy- and apoptosis-related proteins, integrin, proteases, Na Channel protein, PD-L1, VEGFR2, ctDNA, sRNAs, and DNA were used to diagnose and predict various diseases such as lupus nephritis, sepsis, cancers, Parkinson’s disease, traumatic encephalopathy syndrome, hypertension, renal fibrosis, chronic kidney failure, tuberculosis infection, irritable bowel disease, intracerebral hemorrhage, preeclampsia, epilepsy, vasculitis, systemic autoimmune diseases, and acute ischemic stroke. Note fusion expression of exosomal proteins such as EML4-ALK, HER2-HER3, CD9-VEGFR2 were used to develop the diagnostic strategy of cancers.

Among the 18 completed clinical trials, only one (NCT02862470) published their findings referring to urinary exosomal biomarkers in thyroid cancer (Huang et al., 2020). Urinary exosomes from 200 ml human urinary sample were isolated using Ultra 15-centrifugal filters from Millipore, United States and ExoQuick-TC from System Biosciences, United States, and then stored at −80°C after addition of EDTA-free protease inhibitor Cocktail until multiple reaction monitor analysis. Urinary exosomal thyroglobulin (U-Ex Tg) was identified as a proinflammatory predictor and biomarker of thyroid cancer recurrence.

### 2.2 Specific Targeting

EVs of different cellular origins bind to specific recipient cells. For example, exosomes are designed to express Lamp2-RVG on the surface of exosomes, thereby targeting the central nervous system (CNS), and these exosomes contain siRNAs that specifically remove target genes in the CNS after systemic administration (Alvarez-Erviti et al., 2011). Differential and selective protein packaging also occur between cancers of different origins with specific metastatic tropisms, and the packaging of distinct integrins in exosomes from different cancer types plays an important role in determining which organs take up tumor exosomes (Wortzel et al., 2019). Endogenous tumor-derived exosomes can interact with host immune cells and epithelial cells locally and systemically (Chen H. et al., 2021). Tumor-derived exosomes, for example, exosomes released from bladder cancer drive the creation of tumor microenvironment. Preventing the generation or secretion of exosomes inhibited the creation of the immunosuppressive tumor microenvironment (Jiang et al., 2021). Exosomes can target and modify stromal and stem cells in the bone marrow (Gargiulo et al., 2019; Hu et al., 2021). CXCR4+ exosomes selectively accumulated in the bone marrow and thus fusion of CXCR4+ exosomes with liposomes carrying antagomiR-188 promoted ontogeny (Hu et al., 2021). Special membrane surface molecules such as integrin-associated CD47 enhanced the targeting ability and *in vivo* immune escape ability of exosomes from breast cancer cells (Zhao et al., 2020). The use of the membrane protein CD81 in EVs showed improved uptake of exosomes into laminin-secreting mammalian cell lines (Vogt et al., 2021). MSCs-derived exosomes could shift the macrophage polarization state from M1 toward the M2 phenotype *via* transferring the circBCRC-3, which might aid in treatment of the myocardial ischemia-reperfusion injury (SONG et al., 2020). The mechanism of EVs targeting is useful for understanding physiological and pathological processes in diseases, such as development, canonical cellular defenses, and retraining immune and stromal cells towards a pathogenic phenotype.

### 2.3 Circulatory Stability

EVs are resilient lipid bilayer vesicles that can withstand lyophilization (Akers et al., 2016). Exosomes are autologously produced with highly stable components *in vivo* and can be retained in the circulatory system for a longer period compared to liposomes. The distribution of exosomes is closely related to their biological effects. Intravenously injected exosomes rapidly disappear from the circulation and accumulate in the liver, spleen and lungs (Takahashi et al., 2013; Morishita et al., 2015; Yamashita et al., 2018). Small EVs were most abundant in the liver after administration in animals, whereas large EVs were most abundant in the lungs in the first hour and in the liver after 2 h after administration (Kang et al., 2021). The bioactivity of the EVs depends largely on the EV integrity that if its integrity is determined to have been compromised, the EVs lose their biological effects. In most cases, macrophages in the reticuloendothelial system rapidly take up most of the injected exosomes, regardless of cellular origin and style of administration (Charoenviriyakul et al., 2017), which may limit the extracellular exosomes available in recipients. Transmembrane proteins such as CD47 and its signal regulatory protein alpha (SIRPα) prevent exosomes from being phagocytosed by macrophages, thereby increasing the level of exosomes in the blood and improving the delivery of cargos to the target site for therapy (Kamerkar et al., 2017; Cheng et al., 2021). Exosomes with SIRPα can protect themselves from phagocytosis by macrophages, enhancing their biological stability (Koh et al., 2017). PEGylation of exosomes is able to improve their *in vivo* stability, circulation half-lives (Emam et al., 2021). Therefore, chemical modification of EVs is a promising tool used to reduce clearance rates and improve their circulating stability. Although EVs protect their contents from degradation by nucleases and proteases, the circulation stability and half-life of biomarkers were further prolonged by their content and surface modification. A throughout understanding of proteins and specific lipids on EVs surface is needed.

### 2.4 EVs Actively Cross Biological Barriers

The major difficulties faced by conventional synthetic drug delivery systems is their inability to effectively cross biological barriers, including tissue barriers, cellular barriers and intracellular barriers. EVs have the ability to effectively cross these biological barriers (El Andaloussi et al., 2013; Chen et al., 2016). At the tissue level, exosomes have been shown to cross the BBB, one of the most challenging barriers to therapeutic agents, exhibiting great therapeutic potential in cerebrovascular and neurodegenerative diseases (Xu et al., 2021). The BBB is a major barrier to the functional delivery of drugs for the treatment of CNS disorders, as it restricts the passage of almost 98% of small molecule drugs. EVs originating from the CNS have now been shown to cross the BBB and act as drug delivery vehicles for specific neurological patients (Shi et al., 2019). Exosomes are involved in intercellular communication between neuronal cells, and have been shown to protect neuronal integrity, participate in synaptic plasticity, and maintain the brain microenvironment (Nakano and Fujimiya, 2021; Picca et al., 2022). Moreover, exosomes from breast cancer cells induced brain metastasis by impairing the cell-cell junction protein ZO-1 in ECs, which led to increased BBB permeability, and promoted angiogenesis by activating STAT3 and NF-κB in the brain stroma through exosomal Annexin II (Zhou et al., 2014; Maji et al., 2017; Chaudhary et al., 2020). Brain astrocyte-derived exosomes induced metastatic conversion in tumor cells through the transfer miRs targeted by PTEN, resulting in increased brain metastasis (Zhang et al., 2015). Exosomal circNFIX was upregulated in the serum of temozolomide-resistant patients, which promoted tumor growth in glioma cells *in vivo* (Ding et al., 2020). MSC-derived exosomes reduced cerebral vascular ROS production, BBB dysfunction and brain ischemic injury (Pan et al., 2020). MSC-derived exosomes improved the cognitive function of mouse with demyelinating disease (Zhang et al., 2022). Tumor growth factor (TGF)-β1-mediated exosomes derived from non-small cell lung cancer destroyed tight junctions and the integrity of endothelial monolayers and the blood-brain barrier in mice *via* lnc-MMP2-2, promoting brain metastasis of non-small cell lung cancer (Wu D. et al., 2021). Overall, EVs possess ability to actively penetrate biological barriers. Although the underlying mechanism remains unclear (Heidarzadeh et al., 2021) and how to control the penetration of EVs in biological barriers remains a mystery, EVs are promising to be used as drug delivery vehicles for central nervous system diseases. Noteworthy, abnormal cell-derived EVs should be removed to avoid cancer risk.

### 2.5 High Efficient Drug Delivery Vehicles and Anticancer Vaccines

The advantages of MSC- or milk-derived EVs as carriers over synthetic drug delivery systems are their lower immunogenicity and cytotoxicity and better bioavailability and biocompatibility (Tang et al., 2021). The most widely studied carriers in synthetic drug delivery systems are liposomes. Systemic toxicity is one of the factors limiting the clinical application of liposomes (Palazzolo et al., 2018). Synthetic lipid nanoparticles cause toxic immune responses *in vivo* and accumulate mostly in the liver, making them underperform. Moreover, liposomes still present many obstacles in drug delivery to target organs. For example, the use of liposomes activates an acute hypersensitivity reaction, the pseudo-allergic reaction associated with complement activation (Sercombe et al., 2015). However, drug-laden EVs can improve the efficacy of drugs (Hadla et al., 2016). M1 macrophages-derived exosomes have been used as carriers to deliver anticancer drug paclitaxel to tumor tissues by providing a local inflammatory environment (Wang et al., 2019). Endothelial exosomes could transport anticancer drugs such as doxorubicin and paclitaxel across the BBB, exerting a cytotoxic efficacy in brain cancers (Yang et al., 2015). The ability of exosomes to target cancer cells is significantly higher (>10-fold) compared to liposomes of the same size, most likely due to specific ligand-receptor interactions on recipient cells and an optimized endocytosis mechanism (Smyth et al., 2014). Humans can take up exosomes from bovine milk and transfer these exosomes across biological interfaces to surrounding tissues (Kusuma et al., 2016). Paclitaxel-loaded milk exosomes administered orally inhibited tumor growth in the tumor-bearing mouse model without any adverse effects on systemic toxicity and immune responses (Agrawal et al., 2017). Milk exosomes engineered with hydrophilic polyethylene glycol (PEG) coating improved their integrity in acidic gastric environments and mucus permeability by 3 folds compared to unmodified exosomes (Warren et al., 2021). Bovine milk exosomes alleviate cardiac fibrosis *via* enhancing angiogenesis in rats (Zhang et al., 2021). Exosomes carrying curcumin inhibited the growth of lung cancer cells *in vitro* without any toxic effects on healthy cells (Aqil et al., 2017). Exosomes derived from human MSCs have been infused intravenously in rabbits, guinea pigs and rats with no adverse effects on liver or renal function (Sun et al., 2016). EVs secreted by MSCs have shown exceptional immunosuppressive capacity, which has been developed in the clinic for the treatment of autoimmune and inflammatory diseases (ex. SARS-Cov-2) (Bulut and Gürsel, 2020). On the other hand, CD47-mediated immune evasion of exosomes has been demonstrated (Cheng et al., 2021), but the mechanism of immune escape of exosomes is not completely known.

Exosomes released from antigen-presenting cells (APCs), dendritic cells (DCs), and immunogenically dying tumor cells should be used for vaccine development because the surface binding proteins of exosomes are derived from the plasma membrane of their cells of origin (Montaner-Tarbes et al., 2021; Santos and Almeida, 2021) and their immunoprevention ability (Kanlikilicer, 2022). Exosomes from DCs have been shown to have the ability to stimulate immune responses in cancer immunotherapy with comparable effects to parental DCs. Thus, exosomes have been used as a cell-free alternative to DC vaccines in cancer immunotherapy. In addition, nanoparticles have been exploited to deliver immunostimulants such as agonists of toll like receptors (TLRs) to cancer cells *via* lymphatics (Olmeda et al., 2021). Also, exosomes derived from immunogenically dying tumor cells have been exploited as nanovaccines for immunotherapy (Zhou et al., 2022). EVs derived from chimeric antigen receptor (CAR)-T cells expressed lower cytokine levels than CAR-T cells in stimulated tumor cells (Aharon et al., 2021). Overall, EVs, a natural drug delivery vehicle, can overcome the disadvantages of liposomal formulations and have a great potential for drug delivery as an alternative to cell-based therapies.

### 2.6 Oxidative Phosphorylation Capacity

MSC-derived exosomes increased ATP level and reduced oxidative stress to enhance the cardiac function of myocardial ischemic/reperfusion injured mice (Arslan et al., 2013). MSCs-derived exosomes express functional respiratory complexes, consuming oxygen and thus displaying an aerobic respiratory ability independent of whole mitochondria to rescue bioenergetics of injured cells (Panfoli et al., 2016). In particular, ATP synthesis was only detectable in exosomes released from cultured MSCs from human umbilical cord of preterm (28-to 30-weeks gestational age) infants, but was impaired in those from term (≥37-weeks) infants (Panfoli et al., 2016). EVs from cultured MSCs from human umbilical cord of preterm (<34-weeks) infants and term (≥37-weeks) infants consumed oxygen, and express ATP synthase and cytochrome oxidase, but only preterm EVs synthesized ATP (Bruschi et al., 2018). Thus, EVs and exosomes from MSCs from preterm infants synthesized ATP. EVs consume oxygen, but only immature EVs synthesize ATP. Furthermore, urinary exosomes perform oxidative phosphorylation, being able to consume oxygen to aerobically synthesize ATP (Bruschi et al., 2016). EVs (not exosomes) derived from hCMEC/D3 brain ECs increase mitochondrial function in recipient brain ECs exposed to oxygen-glucose deprivation by transferring polarized mitochondria (D'Souza et al., 2021). In the ischemia brain, lack of oxygen leads to mitochondrial dysfunction and subsequent reduction in ATP levels in ECs. EV components such as mitochondria/mtDNA can increase cellular energetics in the recipient hCMEC/D3 cells (Dave et al., 2021). Restoring the cellular ATP in those injured ECs might contribute to the protection of BBB. Overall, EVs might synthesize ATP to repair the injured recipient cells.

### 2.7 Enzyme Activity

EVs contain various proteases including the component of the proteasome (Sanderson et al., 2019). Active 20S proteasome was enriched in platelet-derived EVs, presented in healthy donor blood, and augmented after immune complex injections in mice to contribute to adaptive immunity (Marcoux et al., 2021). 20S proteasome was also enriched in EVs from human donor blood cultured with the malaria parasite *Plasmodium falciparum*, which modulate the mechanical properties of human RBCs by remodeling their cytoskeleton (Dekel et al., 2021). Proteomics analysis revealed that ubiquitin-specific protease 29 (USP29) was increased in EVs derived from MSCs pretreated with melatonin, contributing to microglia/macrophages polarization (Liu W. et al., 2021). In addition, there are various metabolic enzymes in EVs, such as pyruvate dehydrogenase complex, tricarboxylic acid enzymes, electron transport chain enzymes, glycolysis enzymes, fatty acids β-oxidation enzymes, and malate-aspartate enzymes, which are closely linked with energy metabolism (Sakowicz-Burkiewicz et al., 2021). MSC-derived EVs are enriched in tissue inhibitor of metalloproteinases-1 (TIMP-1), CD73 (a GPI-anchored ecto-5′-nucleotidase), and CD39 (ectonucleoside triphosphate diphosphohydrolase-1), which inhibit angiogenesis targeting both extracellular matrix remodeling and endothelial cell migration (Angioni et al., 2020). High level of TNFα converting enzyme (TACE)/ADAM10 presented in plasma EVs of melanoma cancer and HIV-1-infected patients, which is able to stimulate TNFα release from target cells (Lee et al., 2013). The G361 human melanoma cell line-derived exosomes displayed membrane-type 1 matrix metalloproteinase (MT1-MMP) on their surface, which could degrade the extracellular type I collagen (Hakulinen et al., 2008). Heparanase was detected on the surface of exosomes from myeloma patients exposed to bortezomib, which can degrade heparan sulfate present within the extracellular matrix (Bandari et al., 2018). The main apurinic/apyrimidinic (AP)-endonuclease of the DNA base excision repair pathway, the AP endodeoxyribonuclease 1 (APE1) can undergo proteolytic cleavage within exosomes from hepatocellular carcinoma cells (Mangiapane et al., 2021). Thus, EVs from normal cells or healthy donor body fluids possess enzymes, the pathological process or abnormal conditions/stimulation might augment or activate the enzymes in EVs and subsequently induce cytoskeleton remodeling, extracellular matrix remodeling, and biological functions of target cells. In this view point, the addition of protease inhibitors is needed to avoid protein degradation in EV isolations during storage. However, it is unclear whether protease inhibitors will destroy the active components in EVs.

## 3 Challenges

The heterogeneity of the EVs themselves, the lack of isolation and purification standards, optimal storage conditions and the pharmacokinetics or biodistribution patterns are major obstacles to the development of exosomal agents. The possibility of thrombosis and hemostatic perturbations, alloimmune responses and elimination of EVs by the reticuloendothelial system may also limit the application of EVs *via* the systemic route (Rezabakhsh et al., 2021; Table 1).

**TABLE 1:** Possible challenges and required criteria related to clinical application of EVs.

Criteria	Challenges
Isolation and purification techniques	Isolated the contamination EVs with virions and other infectious particles different isolation and purification protocols in different applications, even the same application by different groups lack of evaluation standard for the quality of EVs (such as integrity, concentration, stability, safety, regulatory responses)
Storage	Leakage of content with storage time and temperature possible damage of EVs by freezing and thawing
Drug delivery (pharmacokinetics and biodistribution)	Altered or contaminated exosomal cargos with culture proteins by passage, seeding densities, glucose conditions, and antibiotics in culture medium when cells used as a drug source lack of the feasibility road map to extrapolate the EVs dose for patients from preclinical models biodistribution patterns of EVs varied with injection route, and disease conditions possibility of thrombosis and hemostatic perturbations, and alloimmune responses and elimination of EVs by the reticuloendothelial system

### 3.1 Isolation and Purification Techniques

As shown in Supplementary Table S1, for identifying biomarkers, high throughput technologies including transcriptome, metabonomics, functional proteomics reverse phase protein array, *in situ* proximity ligation assay miRNA-sequencing, proteomes, microfluidic chip, mass spectrometry and recombinant antibody library techniques were carried out on circulating exosomes mainly from blood, urine, and cell culture medium. Yet the minimum volume (>30 ml) of plasma/serum met by ultracentrifugation is commonly hard to yield in clinical trials, although it can isolate the exosomes with high purity. Among the 61 trails, only two state ultracentrifugation will be respectively used for EV isolation from plasma and urine. Currently, there is no uniformity regarding EV isolation methods, and current recommendations are to select different isolation media for use, clinical data collection, and content analysis (Théry et al., 2018). There was extreme variability in the methodology between studies (Kang et al., 2021). The difficulty of isolating and purifying EVs has hindered their use in clinical practice.

The study of EVs of humoral origin is a challenge because the EVs to be isolated come from many different cell types and because of contaminants that have to be removed during EV isolation. However, contamination of the EVs with virions and other infectious particles may not be able to be isolated using current isolation procedures. Currently, ultracentrifugation is the most commonly used technique, which however requires ultra-high centrifugation speeds of up to 1,00,000 g. Ultracentrifugation is complex and time-consuming, and the technique still suffers from several drawbacks, including low reproducibility, low RNA yields, potential damage to EVs, and low sample throughput. Microfluidic-based exosome isolation techniques have been developed to do fast and efficient collection of exosomes (Yang et al., 2017). Five different methods including precipitation (ExoQuick ULTRA), membrane affinity (exoEasy Maxi Kit), size-exclusion chromatography (qEVoriginal), iodixanol gradient (OptiPrep), and phosphatidylserine affinity (MagCapture) rendered exosome samples of different morphology, particle size, and proteomic profile (Veerman et al., 2021). Indeed, EV purification techniques such as immunoaffinity capture, size exclusion, polymer precipitation, differential ultracentrifugation and microfluidic techniques are not always mutually exclusive, and their combined use may enhance the effectiveness of isolation and purification (Stam et al., 2021). Using ultra-high centrifugation to enrich exosomes, followed by a size-exclusion column could effectively remove soluble proteins and other contaminants (Koh et al., 2018), and also improve the proteomics profiling of plasma exosomes (Alameldin et al., 2021). Circulating tumor cells and exosomes could isolate by using microfluidic device with tumor-specific antibodies (Kang et al., 2020). Thus, a more complex procedure of EV isolation and purification might be developed.

The integrity and composition of EVs were impacted by processing methods. Pasteurization or ultra-heat treatment affected EV integrity, reduced their molecular composition including protein signature and RNAs (Kleinjan et al., 2021). In addition, the miR profiles of exosomes obtained with different isolation methods differ (Ding et al., 2018). Exosome-encapsulated miRs in breast cancer can be used as biomarkers for the early diagnosis of breast cancer, but miR studies have failed in the clinical setting due to the lack of standardized methods for the specific purification of exosomes.

Culture conditions affect the content of extracted EVs. In almost current all studies, de-EVs sera are used. Safety and regulatory responses are of great importance to carry out EV-based therapies (Lener et al., 2015). This challenge is highlighted in the MSC-derived EVs driven by the requirement of transition towards clinical application (Witwer et al., 2019). To mitigate the challenge of the biological complexity of MSC-EV preparations, the spatiotemporal biodistribution of MSC-EVs and their components at the tissue, cellular, subcellular, and molecular levels, biological activity and disease outcomes should be considered (Gimona et al., 2021).

Overall, integrity and bioactive components might be diminished by preparation. How to preserve integrity of EVs and produce replicable EVs with stable, safe, and specific regulatory cargos in well-growth cells with a suitable medium should be explored. Although it is suggested that no method is perfect for all EVs studies (Veerman et al., 2021), the development of standardized methods for the isolation and purification of specific EVs through a comprehensive consideration of type, subtype, volume, budget, and so on, will help the use of EVs in clinical diagnostic evaluation and therapeutic interventions.

### 3.2 Storage

A roadmap towards collection, handling and storage of blood EVs were suggested by the ISEV blood EV workgroup (Clayton et al., 2019). Usually, the plasma from patients was frozen and stored at −80°C until isolation of the EVs (Khandagale et al., 2021; Martens-Uzunova et al., 2021). For short-term storage, EVs were resuspended in PBS and stored at 4°C. To improve the rigor and reproducibility of measurements of EVs in blood, the ISEV blood EV workgroup prepared a list of sample storage conditions including temperature of freezing, additions before storage (protease inhibitors, DMSO, other), duration of storage, storage of plasma or isolated EVs, used after single or multiple freeze-thaw cycles, thawing temperature/conditions, and centrifugation post-thawing.

Studies conducted so far have shown that the most promising storage condition for EVs is −80°C (Wu J. Y. et al., 2021). Urinary EVs isolated by ultracentrifugation and stored in PBS at −80°C for up to 24 months preserved their size, concentration, morphology, transcriptome and protein markers, whereas the GC-rich part of the transcriptome and protein markers were degraded in EVs stored at −20°C (Barreiro et al., 2021). However, the storage conditions for EVs in other biological fluids have a significant impact on their biological efficacy. The storage time and temperature of EVs affect their morphology and size distribution, as well as their protein and RNA contents. For example, exosomes from mouse bronchoalveolar lavage fluid (with an average diameter of 95 nm in fresh samples) stored at +4°C had a 10% increase in diameter and reduced charge density (−17.8 ∼ −22.1 mM at 4°C vs. −34.8 ∼ −32.4 mV at 18°C), while that stored at −80°C had a 25% increase in diameter and significantly reduced charge density (−16.5 ∼ −9.9 mV at 80°C) resulting in the formation of multilamellar structure (Maroto et al., 2017). Protein and RNA contents could be wasted with storage time. By LC-MS/MS, the exosomal proteins from mouse bronchoalveolar lavage fluid in the supernatant at the storage conditions of 4°C and −80°C for 4 days were compared and the results showed that distinct proteins leak from the exosomes at different storage temperatures (Maroto et al., 2017). EVs isolated from cerebrospinal fluid of glioblastoma patients that were lyophilized and stored at room temperature for 7 days showed a 37–43% reduction in the number of EVs, with a decreased abundance of representative miRs (Akers et al., 2016). However, it was shown that representative miR levels could be well preserved at room temperature for up to 7 days (Akers et al., 2016). RNA content or miR levels were reduced by two cycles of freezing and thawing (Akers et al., 2016). Melanoma-associated antigen A (MAGEA)-coated extracellular vesicles can be freezen-thawed twice without losing MAGEA4 in detectable amounts (Reinsalu et al., 2021). In recent, EDTA-free protease inhibitor cocktail was added to voided urine before storage at −80°C and transcriptomics analysis for biomarker discovery in diabetic kidney disease (Barreiro et al., 2020). However, the later work from the same group suggested the addition of protease inhibitors before freezing or pre-clearing of urine is not affected the RNA yield of EVs (Barreiro et al., 2021).

Overall, morphology and contents of EVs derived from bronchoalveolar lavage fluid, cerebrospinal fluid, and culture medium might be changed by storage conditions. Although it is unclear how to avoid morphology change of EVs and their cargos leakage during storage, it is recommended −80°C for long-term storage for transcriptomics, with an addition of protease inhibitors for proteomics and protein biomarker discovery.

### 3.3 Pharmacokinetics and Biodistribution Patterns During Drug Delivery

As mentioned above, EVs have various advantages, especially compositional specificity, specific targeting, circulatory stability, active crossing of biological barriers and specific toxicity and immunogenicity. EVs. Exosomes are a promising delivery vector in clinical trials for tissue-specific delivery of biotherapeutics. To date, more than 4 clinical trials using exosomes as delivery vectors are ongoing (Table 2). Exosomes overexpressing CD24 from engineered cell line has already been used to prevent the COVID-19. However, the benefit and risks in the use of EVs for COVID-19 remain to be determined through quality control, pre-clinical safety and efficacy, rational clinical trial design and proper regulatory oversight (Börger et al., 2020).

**TABLE 2:** List of clinical trials of exosomal drug delivery recorded up to October 2021 (available on https://clinicaltrials.gov/ct2/home).

Clinical trials ID	Status	Study	Conditions	Source	Interventions	Phase
NCT04969172	Active, not recruiting	Overexpressing CD24 to prevent clinical deterioration in patients with moderate or severe COVID-19 infection	COVID-19	Human embryonic kidney T-REx™-293 cells that constitutively express high levels of human CD24	Exosomes overexpressing CD24 (1010 exosome particles)	Phase II
NCT04902183	Recruiting	Exosomes overexpressing CD24 in two doses for patients with moderate or severe COVID-19	COVID-19	Exosomes overexpressing CD24	CovenD24 (Exo-CD24) 10^9 exosome particles	Phase II
NCT04747574	Recruiting	CD24-Exosomes in patients with COVID-19 infection	SARS-CoV-2	T-REx™-293 cells engineered to express CD24	EXO-CD24 10^8, 10^9, or, 10^10 exosome particles/2 ml	Phase I
NCT03608631	Recruiting	iExosomes in treating participants with metastatic pancreas cancer with KrasG12D mutation	KRAS NP_004976.2:p.G12D metastatic pancreatic adenocarcinoma pancreatic ductal adenocarcinoma stage IV pancreatic cancer AJCC v8	KrasG12D siRNA-loaded MSCs-derived exosomes	MSCs-derived exosomes with KRAS G12D siRNA (dose-escalation study)	Phase I

The dose was mainly determined by the particle number of the exosomes. However, the cargo profile of exosomes could be altered or contaminated with the proteins in the culture medium by the passage (Patel et al., 2017; Ludwig et al., 2019), seeding density (Patel et al., 2017; Ludwig et al., 2019), glucose conditions (Rice et al., 2015), and antibiotics (Németh et al., 2017) in culture medium. Compression stress could inhibit osteoblast differentiation *via* altering the exosomal miRNA expression profile *in vitro* (Wang Y. et al., 2021). The role of culture conditions in the heterogeneous composition of exosomes should be carefully considered when cells are used as a source of exosomes. Another challenge is the pharmacokinetic profile of the liposomal drugs depends on their composition and varies by 30% when the dose converted from animal models to humans using the power-log relationship between body weight and drug clearance among mammals, which is presented in the PEGylated liposomes (Caron et al., 2011). The road map is much needed to extrapolate the dose of EVs to patients from preclinical models (Gupta et al., 2021).

EVs could actively cross biological barriers. It should be noted that only a fraction of the dose of EVs has been shown to localize to the central nervous system after systemic administration (Gupta et al., 2021). A systematic review showed that the biodistribution of EVs is body-wide, regardless of the origin or size of EVs, or the species treated, primary to the liver, lungs, kidneys, and spleen (Kang et al., 2021). However, biodistribution patterns of EVs have been rendered by the different EVs injection routes (intravenous, intraperitoneal, and subcutaneous injections) (Wiklander et al., 2015). Inflammation could drive the homing of intranasally administered MSC-derived exosomes into the brain (Perets et al., 2019). A deeper understanding of the mechanism underlying EV uptake in specific diseases is required to reliably predict the biodistribution and therapeutic effects of EVs. Moreover, the purity of EVs influenced their biodistribution (Nordin et al., 2015).

## 4 Summary and Prospects

EVs have advantageous properties including composition specificity, target specificity, circulating stability, active penetration of biological barriers and specific toxicity and immunogenicity. Exosomal content has attracted much attention as a promising diagnostic and prognostic tool of recalcitrant or difficult-to-treat diseases such as neoplastic diseases, and degenerative diseases of the central nervous system. However, due to the complexity of the biochemical properties of the EVs themselves, the lack of standardized isolation and purification methods for EVs, the limited drug delivery efficiency of EVs, the isolation of EVs contaminated with virions and other infectious particles, and insufficient production are still major challenges. Although Electron microscopy, flow cytometry, western blotting, FISH examination and qRT-PCR had been used to identify EVs, how to evaluate the quality of EVs still lacks criteria. Furthermore, the outward leakage of proteins from EVs during storage challenges the consistency for detection of cargos. Sample always stored before analysis, especially in the case of proteomic analysis of subsequent samples in the case of a prospective study of human samples, it would be very important to examine whether it is feasible to use frozen EVs in pre-clinical and clinical studies. Guidelines should be developed to promote standardization in future to identify the biomarkers of EVs, which shall include available information including source, volume, anticoagulant condition, isolation methods, identification, and quantification control, special marker and their detection methods, possibly also pathway or interaction prediction, and diagnostic performances. For drug delivery, additional attentions on storage, pharmacokinetics and biodistribution patterns of EVs/engineered EVs are required.

These have also disturbed the control of qualification and stability of EVs by each manufacturer. There is also litter information on how to fully determine the protein composition of EVs when selecting donor cells for EV production and accurately assess the potential immunogenicity of the EVs used to avoid adverse events in patients *in vivo*. In addition, most existing studies on EVs have been derived from EVs in cell cultures, and few EVs have been extracted directly from patients’ body fluids. Careful consideration needs to be given to how to more effectively and safely validate the results of cell experiments with *in vivo* work. Existing limitations in EVs research will soon be resolved and will accelerate the research into EVs transformation in the future.
