# Supplementary material for: Biological Features of Extracellular Vesicles and Challenges
Source: Front Cell Dev Biol. 2022 Jun 24;10:816698. doi: 10.3389/fcell.2022.816698 (PMC9263222; doi:10.3389/fcell.2022.816698)
Supplement: Supplementary file 1 [file Table1.docx]

**Supplementary Table 1. List of clinical trials of exosomal biomarker (available on https://clinicaltrials.gov/ct2/home)**

| **Clinical trials ID** | **Status** | **Study** | **Conditions** | **Source** | **Measurements** | **Method** |
| --- | --- | --- | --- | --- | --- | --- |
| NCT04894695 | Recruiting | Urine exosomes to identify biomarkers for LN | Lupus nephritis (LN) | Urine | Molecule like miRNAs | Transcriptome and/or metabonomics sequencing of exosomes |
| NCT03222986 | Completed | Sepsis-damaged organs-double-markers identification of organ failure using fluorescent nanoparticle tracking analysis | Sepsis with multiple organ dysfunction (MOD) | Co-culture medium and clinical sepsis specimens | Ubiquitination-autophagy-apoptosis biomarkers | Nanoparticle tracking analysis with double markers identification; exosome proteomics |
| NCT04315753 | Recruiting | Circulating and imaging biomarkers to improve lung cancer management and early detection | Lung cancer | Blood | Exosome antigens | Functional proteomics reverse phase protein array (RPPA) |
| NCT03874559 | Recruiting | [Exosomes in rectal cancer](https://clinicaltrials.gov/ct2/show/NCT03874559?term=exosome+and+biomarker&draw=3&rank=5) | Rectal cancer | Blood draw | Exosomal biomarkers |  |
| NCT04394572 | Recruiting | Identification of new diagnostic protein markers for colorectal cancer | Colorectal cancer | Blood | Macromolecules, integrins, and metallo proteases | Diagnostic performances (sensitivity and specificity) |
| NCT01860118 | Completed | LRRK2 and other novel exosome proteins in Parkinson's disease | Parkinson's Disease | Whole blood samples and urine | LRRK2 expression and/or phosphorylation | exosome-proteomes |
| NCT04523389 | Recruiting | Contents of circulating extracellular vesicles: biomarkers in colorectal cancer patients | Colorectal cancer | Blood | Contents (protein, lipid, RNA ...), size and number of exosomes |  |
| NCT03108677 | Recruiting | Circulating exosome RNA in lung metastases of primary high-grade osteosarcoma | Lung metastases Osteosarcoma | Peripheral blood | Levels and mutations of exosomal RNA, and miRNA | Whole RNA sequencing. |
| NCT05101655 | Enrolling | Construction of microfluidic exosome chip for diagnosis of lung metastasis of osteosarcoma | Osteosarcoma | Peripheral blood | Levels of exosome and its subgroups | Microfluidic chip |
| NCT04227886 | Recruiting | Study on predictive biomarkers of neoadjuvant chemoradiotherapy for rectal cancer | Rectal neoplasm | Tumor tissue and plasm | Gene expression differences | Whole RNA sequencing |
| NCT04120272 | Recruiting | Discovery for biomarkers and risk factors for postoperative delirium in elderly patients with spine surgery | Spinal disease | Urine and blood | Gene expression in exosomes integrative analysis to identify the mRNA-miRNA interaction | SAM tool and DEseq2 Gene ontology (GO) and KEGG databases |
| NCT04164966 | Recruiting | Development of novel biomarkers for the early diagnosis of type 1 diabetes | Type 1 diabetes | Blood | Characterization (the number/concentration) of circulating β-cell specific exosomes |  |
| NCT04323579 | Recruiting | Validation of multiparametric models and circulating and imaging biomarkers to improve lung cancer early detection. | Lung cancer | Blood | The role of molecular and cellular biomarkers (exosomes, protein signatures, circulating tumor cells, microRNA) and radiomic signature | Exosome antigens analysis |
| NCT03985696 | Recruiting | Exosomes and immunotherapy in non-Hodgkin B-cell l  ymphomas | Lymphoma | Cell and blood (EDTA) | Quantification of CD20 and PDL-1 in exosomes |  |
| NCT04928534 | Recruiting | Cohort study of blood biomarkers for TES | Traumatic encephalopathy syndrome (TES) | Blood | RNA biomarkers | Transcriptomics, proteomics |
| NCT04534647 | Recruiting | Serological and urinary biomarkers in Latin American patients with systemic lupus erythematosus | Lupus nephritis | Urine and blood | Biomarkers in urinary exosomes | Transcriptome |
| NCT03267160 | Completed | A study of exosome proteomics and hemodynamics in sepsis | Sepsis with respiratory and heart involvement | Blood and urine | Analyze autophagy, and apoptosis related biomarkers of exosomes | Proteomics |
| NCT03895216 | Recruiting | Identification and characterization of predictive factors of onset of bone metastases in cancer patients | Bone Metastases | Blood | miRNAs and protein changes of circulating tumor exosomes | Next Generation Sequencing (NGS), Triple TOF mass spectrophotometer |
| NCT02226055 | Completed | An Investigation into the cardiovascular risk and aetiology of CKDu in Sri Lanka | CKD of unknown aetiology | Serum and urine | Exosomes as liquid biopsy biomarker |  |
| NCT04556916 | Recruiting | Early detection of prostate cancer | Prostate cancer | Blood (EDTA) | Exosomes |  |
| NCT03034265 | Completed | New biomarkers and difficult-to-treat hypertension | Hypertension | Urine | Repeatability of exosomal Na channel proteins under pontaneous vs. standardized laboratory conditions |  |
| NCT04288141 | Recruiting | A study to measure the expression of the HER2-HER3 dimer in tumour and blood (exosomes) samples from patients with HER2 positive breast cancer receiving HER2 targeted therapies | HER2-positive Breast Cancer | Blood | Exosomal HER2-HER3 dimer expression | FLIM-FRET |
| NCT02862470 | Completed | Anaplastic thyroid cancer and follicular thyroid cancer derived exosomal analysis via treatment of lovastatin and vildagliptin and pilot prognostic study via urine exosomal biological markers in thyroid cancer patients | Thyroid Cancer | Urine | Exosomal proteins |  |
| NCT03280576 | Completed | Validation of progranulin as a biomarker for sepsis | Sepsis | Blood | miRNA expression in exosomes | Next-generation sequencing |
| NCT03870542 | Recruiting | Multicenter, prospective study for urinary exosomal biomarkers of kidney allograft tubulointerstitial fibrosis | Renal Fibrosis, Kidney transplant failure | Urine | Identify biomarkers that represent fibrosis | Proteomics analysis and RNA-sequencing |
| NCT03202212 | Completed | Effect of mixed on-line hemodiafiltration on circulating markers of inflammation and vascular dysfunction | Chronic kidney failure, dialysis related complication | Blood | miRNA in exosomes | Quantitative real-time PCR |
| NCT02921854 | Completed | Detection of circulating biomarkers of immunogenic cell death | Non-small cell lung cancer | Blood | Changes of exosomes |  |
| NCT03941210 | Recruiting | Micro RNA as prediction and/or prognostic markers of IRIS in TB-HIV co-infected patients | HIV infection, Tuberculosis infection | Plasma/cells | miRNA expression profile | Flow cytometry |
| NCT03694483 | Recruiting | Prostasomes as diagnostic tool for prostate cancer detection | Prostate cancer | Blood | Prostasomes purification methodology, miRNA expression profiles of the purified protostomes | ExoPLA (Exosome in situ Proximity Ligation Assay miRNA-sequencing |
| NCT04427475 | Recruiting | Prediction of immunotherapeutic effect of advanced non-small cell lung cancer | Non-small cell lung cancer | Blood | Plasma exosomes PD-L1 and miRNAs |  |
| NCT03264976 | Recruiting | Role of the serum exosomal miRNA in diabetic retinopathy | Diabetic Retinopathy | Blood | Serum exosomal miRNAs | miRNA sequencing |
| NCT04879810 | Recruiting | Plant exosomes +/- curcumin to abrogate symptoms of inflammatory bowel disease | Irritable bowel disease | Ginger | The effect of ginger exosomes on biomarkers of inflammation | PO adminstration |
| NCT04979767 | Recruiting | Function of circulating exosomes in sepsis-induced immunosuppression | Sepsis | Blood | Identify biomarkers of immune status |  |
| NCT05035134 | Recruiting | Application of circulating exosomes in early diagnosis and prognosis evaluation after intracerebral hemorrhage | Intracerebral hemorrhage | Serum | The type and content of serum exosomes | RNA sequencing and proteome |
| NCT03562715 | Completed | microRNAs role in pre-eclampsia Diagnosis | Preeclampsia | Peripheral blood，  umbilical cord MSCs | Comparison of miRNAs 136, 494 and 495 in exosomes of between peripheral blood and cell conditioned media |  |
| NCT03419000 | Recruiting | Circulating microRNAs as biomarkers of RESPIratory dysfunction in patients with refractory epilePSY | Drug resistant epilepsy | Blood  (4 ml in EDTA and dry tubes) | Expression profile of miRNAs in the exosomes |  |
| NCT02823613 | Completed | The influence of high and low salt on exosomes in the urine | Healthy male with high/low salt diet | Urine | Quantification of epithelial sodium channel (ENaC) in urinary exosomes | Ultracentrifugation，western blotting |
| NCT04029740 | Recruiting | Exosomal microRNAs as a biomarker in panic disorder and in response to CBT | Panic disorder | Blood | Differential expression of plasma exosomal miRNA |  |
| NCT04629079 | Recruiting | Improving the early detection of lung cancer by combining exosomal analysis of hypoxia with standard of care imaging | Lung cancer | Blood | Exosomal expression of P4HA1, precursor miRNA |  |
| NCT04053855 | Recruiting | Evaluation of urinary exosomes presence from clear cell renal cell carcinoma | Clear cell renal cell carcinoma | Urine  (100 mL) | Number of patients with CD9+ (exosome marker) and CA9+ (clear cell kidney cancer marker) exosomes; percentage of CD9+/CA9+ exosomes; percentage of CD9+/VGEFR2+ exosomes | Electron microscopy technique, flow cytometry, RT-qPCR |
| NCT03432806 | Recruiting | A study of imaging, blood, and tissue samples to guide treatment of colon cancer and related liver tumors | Colon cancer, liver tumors | Peripheral venous blood (10 mL prior to surgery, 30 mL at the first postoperative clinic visit) | Isolation of at least 5 μg of exosomal protein |  |
| NCT04499794 | Recruiting | The study of exosome EML4-ALK Fusion in NSCLC clinical diagnosis and dynamic monitoring | NSCLC patients | Plasma | Prognostic value of exosome EML4-ALK fusion expression | Exosome fusion detection, and FISH examination |
| NCT04357717 | Recruiting | ExoDx prostate evaluation in prior negative prostate biopsy setting | Prostate cancer | Urine | Clinical evaluation of ExoDx^TM^ Prostate (IntelliScore) |  |
| NCT03334708 | Recruiting | A study of blood based biomarkers for pancreas adenocarcinoma | Pancreatic cancer | Blood | Change in exosomes |  |
| NCT03569566 | Enrolling | Performance determinants factors in elite endurance athletes | Elite endurance athletes | Plasma | The volume and specific types of exosomes, and exosome-cargos | Ultracentrifugation |
| NCT03824275 | Recruiting | 18F-DCFPyL positron emission tomography (PET)/computed tomography (CT) in men with prostate cancer | Prostatic neoplasms | Blood | The characterize ctDNA and exosomes and their correlation |  |
| NCT04530890 | Recruiting | Interest of circulating tumor DNA in digestive and gynecologic/breast cancer | Breast cancer  Digestive cancer  Gynecologic cancer | Blood | Evaluate the diagnostic, prognostic and predictive value of ctDNA and exosomes, detect certain molecular alterations using ctDNA and exosomes |  |
| NCT04636788 | Recruiting | Circulating extracellular exosomal small RNA as potential biomarker for human pancreatic cancer | Pancreas adenocarcinoma | Blood  (Venous, 12 mL) | exo-sRNAs, and their diagnostic and prognostic value | Next-generation sequencing |
| NCT04948437 | Recruiting | Urinary exosomal biomarkers of thyroglobulin and galectin-3 for prognosis and follow-up in patients of thyroid cancer | Thyroid cancer  Papillary thyroid cancer  Follicular thyroid cance | Urine | Exosomal thyroglobulin, galectin-3, calprotectin A9/8, transketolase, keratin 19/8, angiopoietin-1, annexin II, afamin, and tissue inhibitor of metalloproteinase |  |
| NCT04542902 | Recruiting | Non-coding RNAs analysis of eosinophil subtypes in asthma | Allergic asthma  Severe eosinophilic asthma | Blood | ncRNA levels in exosomes |  |
| NCT00331331 | Completed | The vitreous proteome and inflammatory mediators in ocular inflammatory disease | Uveitis, Vasculitis, Ocular inflammatory disease | Vitreous and anterior chamber fluid of an eye | Exosomes |  |
| NCT03984006 | Completed | Early detection of autoimmune thyroid heart disease via urinary exosomal proteins | Thyroid diseases, heart failure | Urine | Exosomal proteins NT-proBNP |  |
| NCT04924504 | Recruiting | Mechanisms behind severe insulin resistance during pregnancy in women with glucose metabolic disorders (SIR-MET) | Women with type 2 diabetes or gestational diabetes | Serum and plasma  (Maternal blood, umbilical cord blood) | Level, content and bioactivity of exosomes | SWATH mass spectrometry  miRNA sequencing |
| NCT02890121 | Completed | Molecular reclassification to find clinically useful biomarkers for systemic autoimmune diseases | Systemic autoimmune diseases | Blood and urine | Gene expression in exosomes |  |
| NCT03944603 | Recruiting | Longitudinal innate immunity and aging study | Healthy older adults ages 60-89 | Blood and cerebrospinal fluid | Innate immune markers in exosomes |  |
| NCT03459703 | Completed | Effect of time-restricted feeding on fat loss and cardiometabolic risk factors in overweight adults | Obesity | Urine | Exosomes (particles/mL) |  |
| NCT04617405 | Recruiting | Hormonal and inflammatory changes during pregnancy in women with glucose metabolic disorders. | Pregnant women with type 2 diabetes or gestational diabetes | Serum and plasma | The level, content and bioactivity of exosomes | SWATH mass spectrometry,  miRNA sequencing |
| NCT03971955 | Recruiting | Characterization of adult onset autoimmune diabetes | Diabetes | Blood | Specific miRNAs in exosomes |  |
| NCT01294072 | Recruiting | Study investigating the ability of plant exosomes to deliver curcumin to normal and colon cancer tissue | Colon cancer | Plant | Delivered curcumin by plant exosomes | Take plant exosomes orally |
| NCT03911999 | Completed | Exosomal microRNA in predicting the aggressiveness of prostate cancer in chinese patients | Prostate Cancer | Urine | Exosomal miRNA | Next generation sequencing (NGS) |
| NCT04266639 | Recruiting | Rheo-Erythrocrine dysfunction as a biomarker for RIC treatment in acute ischemic stroke | Acute ischemic stroke | Plasma | Exosome surface makers and content (DNA and RNA including miRNA) | ELISA, western blot, recombinant antibody library techniques, next generation sequencing, and qRT-PCR |
